# Coronary CTA for Surveillance of Cardiac Allograft Vasculopathy

**DOI:** 10.1007/s12410-018-9467-z

**Published:** 2018-09-24

**Authors:** Nishant R. Shah, Ron Blankstein, Todd Villines, Hafiz Imran, Alan R. Morrison, Michael K. Cheezum

**Affiliations:** 1 Lifespan Cardiovascular Institute, Division of Cardiovascular Medicine, Dept. of Medicine, Brown University Alpert Medical School, Providence, RI USA; 2 Dept. of Medicine (Cardiovascular Division) and Radiology, Brigham and Women’s Hospital, Harvard Medical School, Boston, MA USA; 3 Dept. of Medicine, Cardiology Service, Walter Reed National Military Medical Center, Bethesda, MD USA; 4 Dept. of Medicine, Cardiology Service, Fort Belvoir Community Hospital, Ft. Belvoir, Fairfax County, VA USA

**Keywords:** Cardiac allograft vasculopathy, Coronary CT angiography, Dual-source CT, Multisegment reconstruction, Intracycle motion correction

## Abstract

**Purpose of Review:**

The purpose of this review is to highlight recent hardware and software advances in coronary computed tomography angiography (CTA) that make it a potentially viable alternative to invasive coronary angiography for surveillance of cardiac allograft vasculopathy (CAV) in heart transplant recipients.

**Recent Findings:**

Dual-source CT, multisegment reconstruction, and intracycle motion correction algorithms are all technologies applied during or after image acquisition that can improve image quality and diagnostic accuracy in patients with elevated heart rates, such as heart transplant recipients. CT fractional flow reserve may also add value in this clinical scenario.

**Summary:**

Coronary CTA now has equivalent diagnostic accuracy, offers more nuanced anatomic information, is inherently safer, and could be less costly than invasive coronary angiography. For these reasons, coronary CTA may now be a viable alternative to ICA for CAV surveillance in heart transplant recipients.

## Introduction

**Fig. 1:**
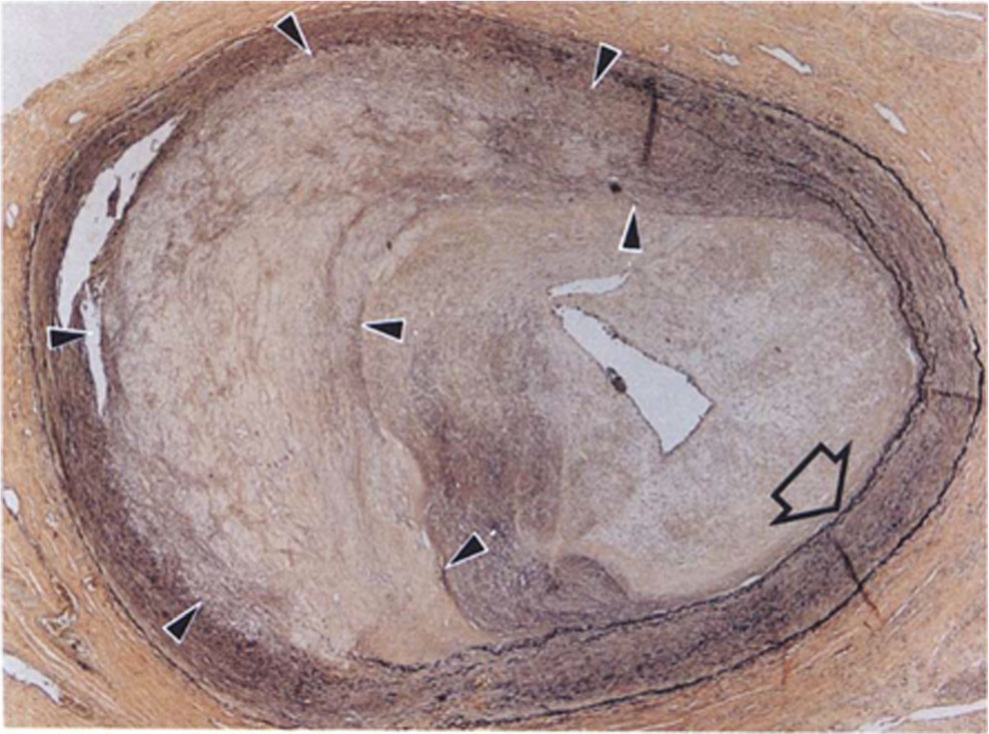
Cardiac allograft vasculopathy (CAV) superimposed on asymmetric native coronary artery disease (CAD; arrowheads). The elastic lamina (open arrow) is intact beneath the CAV lesion but not the CAD lesion. Used with permission from Schoen & Libby, Trends Cardiovasc Med. 1991 Jul-Aug; 1 [5]:216–23

Cardiac allograft vasculopathy (CAV) occurs within 5 years in nearly 30% of adult heart transplant recipients and is associated with significant morbidity and mortality [1]. CAV is a delayed-type hypersensitivity immune response that results in coronary artery intimal hyperplasia and typically manifests as diffuse, concentric luminal narrowing and ischemic graft failure [2, 3]. This pathophysiology is distinct from the asymmetric lipid-rich plaque formation seen in non-transplanted patients with traditional coronary artery disease (CAD). However, as shown in Fig. 1, CAV often manifests concurrently with CAD in heart transplant recipients [4].

A standardized nomenclature for CAV was adopted by the International Society for Heart and Lung Transplantation (ISHLT) in 2010 and remains the most widely used classification in clinical practice [5]. In delineating mild, moderate, and severe CAV, this classification relies primarily on the location and severity of stenoses on invasive coronary angiography (ICA) but also incorporates echocardiography-based measures of systolic dysfunction and restrictive physiology as potential surrogate manifestations of subclinical and microvascular CAV. Accordingly, the most recent ISHLT guidelines for the care of heart transplant recipients recommend surveillance for CAV in all patients with annual or biannual ICA and resting echocardiography [6••].

### Limitations of Invasive Coronary Angiography for CAV Surveillance

**Fig. 2:**
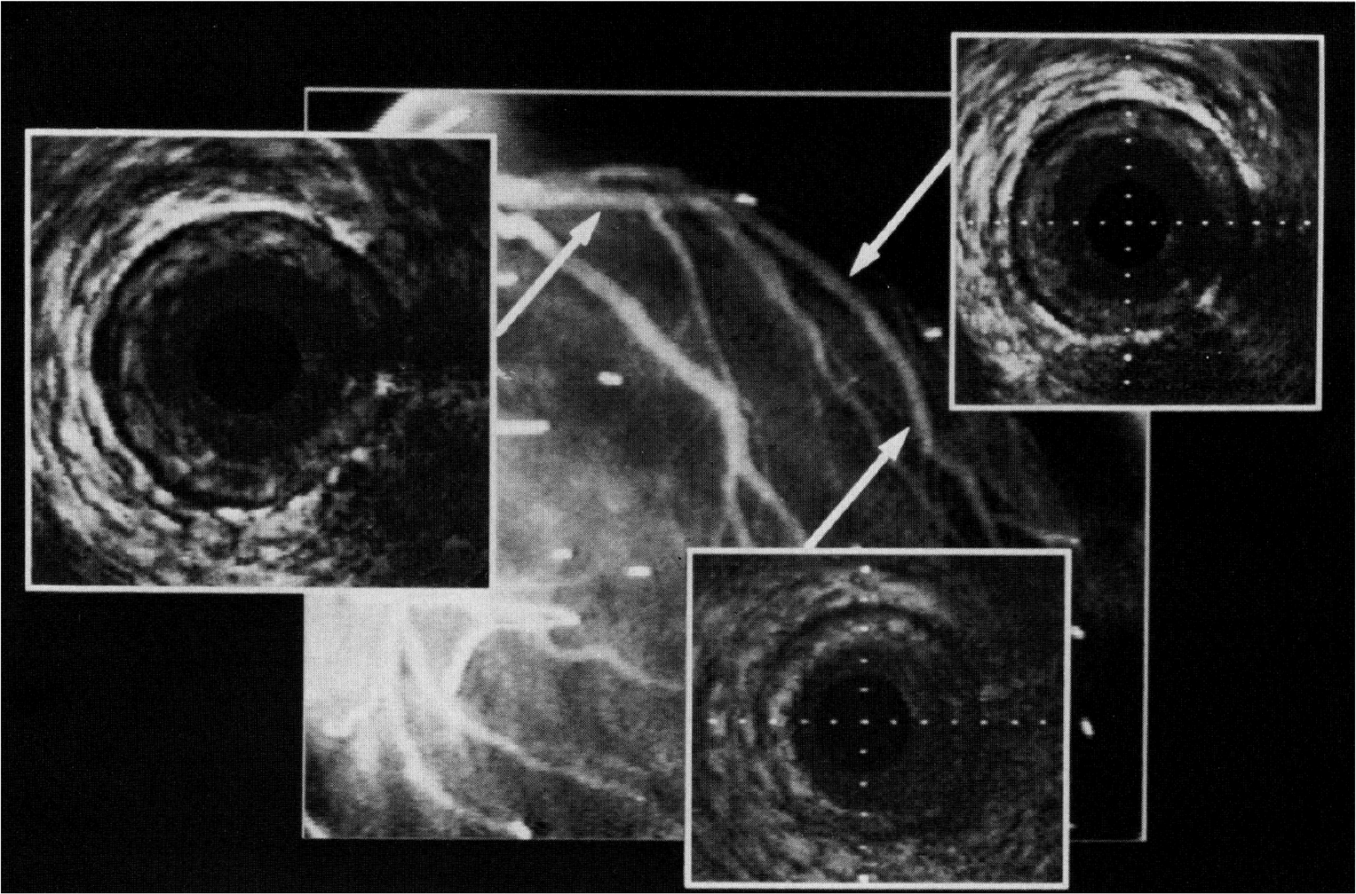
Invasive coronary angiogram with intravascular ultrasound images from three representative sites in the left anterior descending coronary artery (LAD) of a 37-year-old man 3 years after heart transplantation. Although the angiogram is without evidence of coronary disease, the ultrasound images demonstrate severe concentric intimal thickening throughout the proximal and mid LAD. Used with permission from St. Goar et al. Circulation. 1992 Mar;85 [3]:979–87

While ICA is currently recommended for first-line CAV surveillance in heart transplant recipients, the ISHLT and others have acknowledged shortcomings associated with this strategy. First, the sensitivity of ICA is dependent on definition of a normal-caliber reference lumen against, which a presumed stenotic segment is relatively compared. However, diseased coronary arteries with diffuse, concentric CAV beginning at the ostium may not have a normal-caliber reference, and thus are more likely to be falsely diagnosed as normal. Clinically, this discrepancy can be strikingly evident when comparing same-vessel ICA and intravascular ultrasound (IVUS) images. Figure 2 shows one such example in a heart transplant recipient, where ICA of the left anterior descending (LAD) coronary artery is essentially normal in the presence of severe concentric intimal thickening seen on IVUS [7]. Adjunctive pressure wire-based invasive measurements, including fractional flow reserve (FFR), coronary flow reserve (CFR), and index of microcirculatory resistance (IMR) can improve the diagnostic accuracy and prognostic value of ICA performed for CAV surveillance [8, 9] but must be performed in all three coronary artery territories for complete assessment. These techniques therefore involve significantly higher procedural risk, time, and resources compared to ICA alone.

**Fig. 3:**
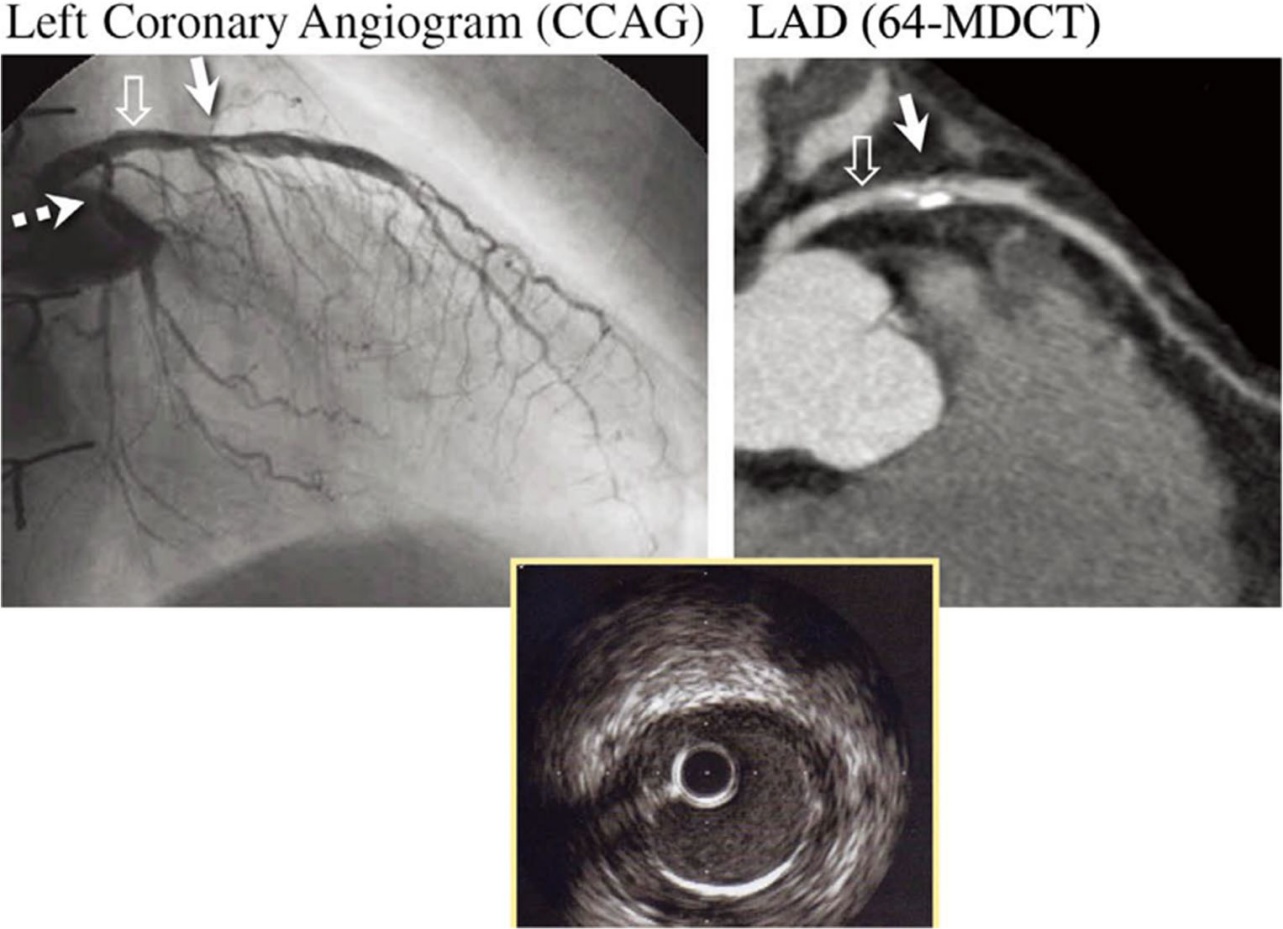
Coronary stenosis in the proximal–middle LAD (solid arrows) revealed by both invasive and CT coronary angiography in a heart transplant recipient. Intravascular ultrasounds performed simultaneously confirmed intimal hyperplasia in the proximal LAD (open arrows). Used with permission from Nunoda et al. Circ J. 2010 May;74 [5]:946–53

Second, even when luminal narrowing is recognized, ICA alone does not identify plaque characteristics that may identify patients with an increased risk of future adverse cardiac events (i.e., positive remodeling, low attenuation plaque, spotty calcification) [10]. Figure 3 shows an example in a heart transplant recipient, where ICA demonstrates narrowing of the LAD coronary artery, and coronary CTA provides additional characterization of the calcified and noncalcified plaque burden with positive remodeling. Figure 3 also shows that IVUS in the proximal LAD revealed intimal thickening that is not particularly evident on either of the angiography images [11]. While IVUS and optical coherence tomography can clearly provide plaque characterization during ICA, they must be performed in all three coronary artery territories for complete assessment, and therefore, involve significantly higher procedural risk, time, and resources compared to ICA alone.

Finally, ICA is associated with small but measurable risks inherent with invasive procedures [12]. Use of radial artery access, as opposed to femoral artery access, reduces but does not eliminate vascular access site complications [13]. Many of the assumed risks associated with ICA are non-existent or clinically negligible for non-invasive diagnostic modalities like coronary CTA. Importantly, this differential in assumed risk is additive each time a heart transplant recipient undergoes ICA as opposed to non-invasive testing.

### Coronary CTA for CAV Surveillance: Heart Rate-Related Challenges

The 2010 ISHLT guidelines explicitly acknowledge the “promise” of coronary CTA for evaluation of CAV in heart transplant recipients but also state that higher resting heart rates in these patients limit its technical quality [6••]. Resting tachycardia is indeed a common finding in heart transplant recipients, whose new hearts are denervated and therefore lack any vagus nerve-mediated reduction in automaticity of the sinoatrial node. The major challenge with high heart rates during coronary CTA is increased occurrence of motion artifacts, such as blurring, ghosting, winging, or streaking [14]. In addition, high heart rates exacerbate streak artifacts associated with dense materials such as calcium. For this reason, current guidelines from the Society of Cardiovascular Computed Tomography (SCCT) state that optimal image quality for coronary CTA is reliably achieved when the patient has a low heart rate (≤ 60 bpm) and a regular rhythm during acquisition, although higher heart rates may be acceptable depending on the scanner [15]. Typically, the use of beta-blockers, calcium channel blockers, and ivabradine have less effect in heart transplant recipients. In these patients, advancements in CT technology now permit diagnostic image quality at higher heart rates.

### Recent CT Technologies Allowing for Higher Heart Rate Acquisitions

#### Dual-Source CT (DSCT)

**Fig. 4:**
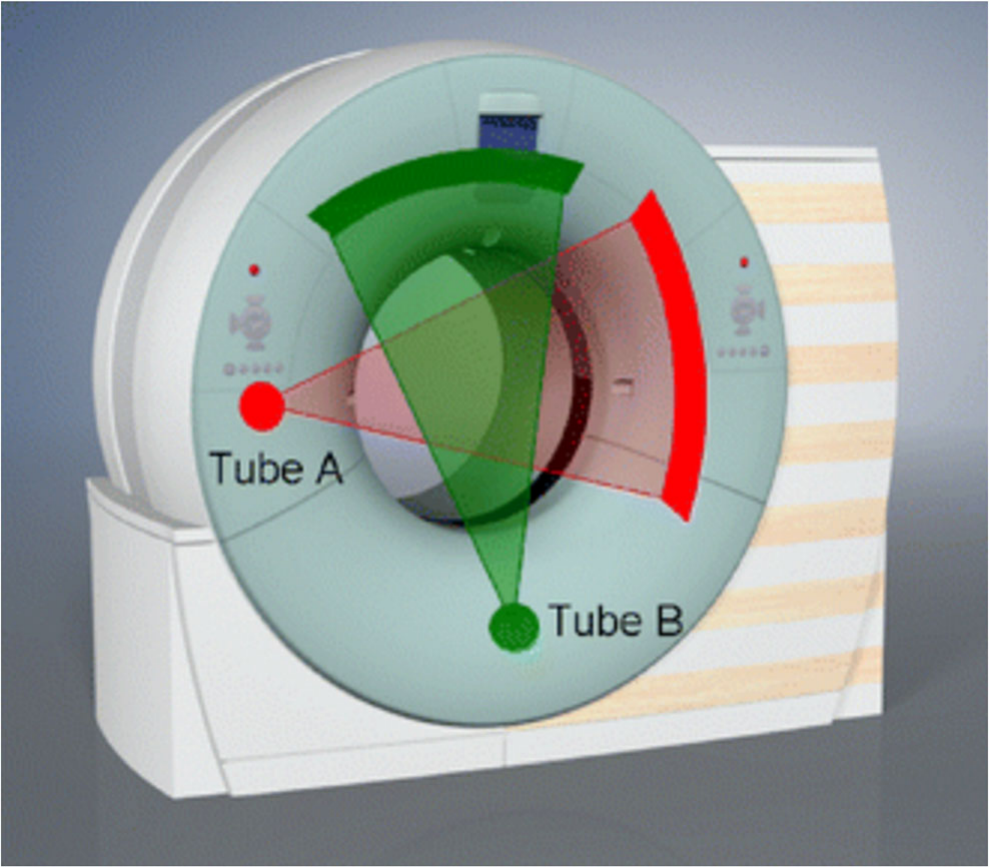
Dual-source computed tomography (DSCT) system with a schematic illustration of the acquisition principle using two tubes and two corresponding detectors offset by 90°. A scanner of this type provides temporal resolution equivalent to a quarter of the gantry rotation time independent of the patient’s heart rate. Used with permission from Flohr et al. Eur Radiol. 2006 Feb;12 [2]:256–68

As shown in Fig. 4, DSCT employs two x-ray tubes and two corresponding detectors offset by 90° to each other to improve temporal resolution to 83 ms [16]. By comparison, conventional coronary CTA typically achieves a temporal resolution of 135–200 ms, requiring acquisition over 5–10 s and higher radiation exposure. This is notable when considering optimal windows for coronary artery imaging, as the temporal resolution needed to obtain motion-free diagnostic image quality must fall within the isovolumetric relaxation time (IVRT, 80–120 ms) or diastasis (≤ 300 ms). Of these, IVRT is constant and heart rate independent, while diastasis is diminished with increasing heart rates. Thus, when temporal resolution falls within the motion-free IVRT window, optimal image quality may be obtained at increased heart rates. This perhaps explains why in patients with heart rates > 80 beats per minute (bpm) who receive no beta-blocker premedication prior to DSCT imaging, image quality with late systole image reconstruction is typically improved when compared to late diastole [17••]. Using these modified image reconstruction protocols, DSCT in patients with heart rates > 65 bpm offer similar diagnostic accuracy compared to lower heart rates for the detection of coronary artery stenosis as assessed by ICA [18, 19].

#### Multisegment Reconstruction (MSR)

**Fig. 5:**
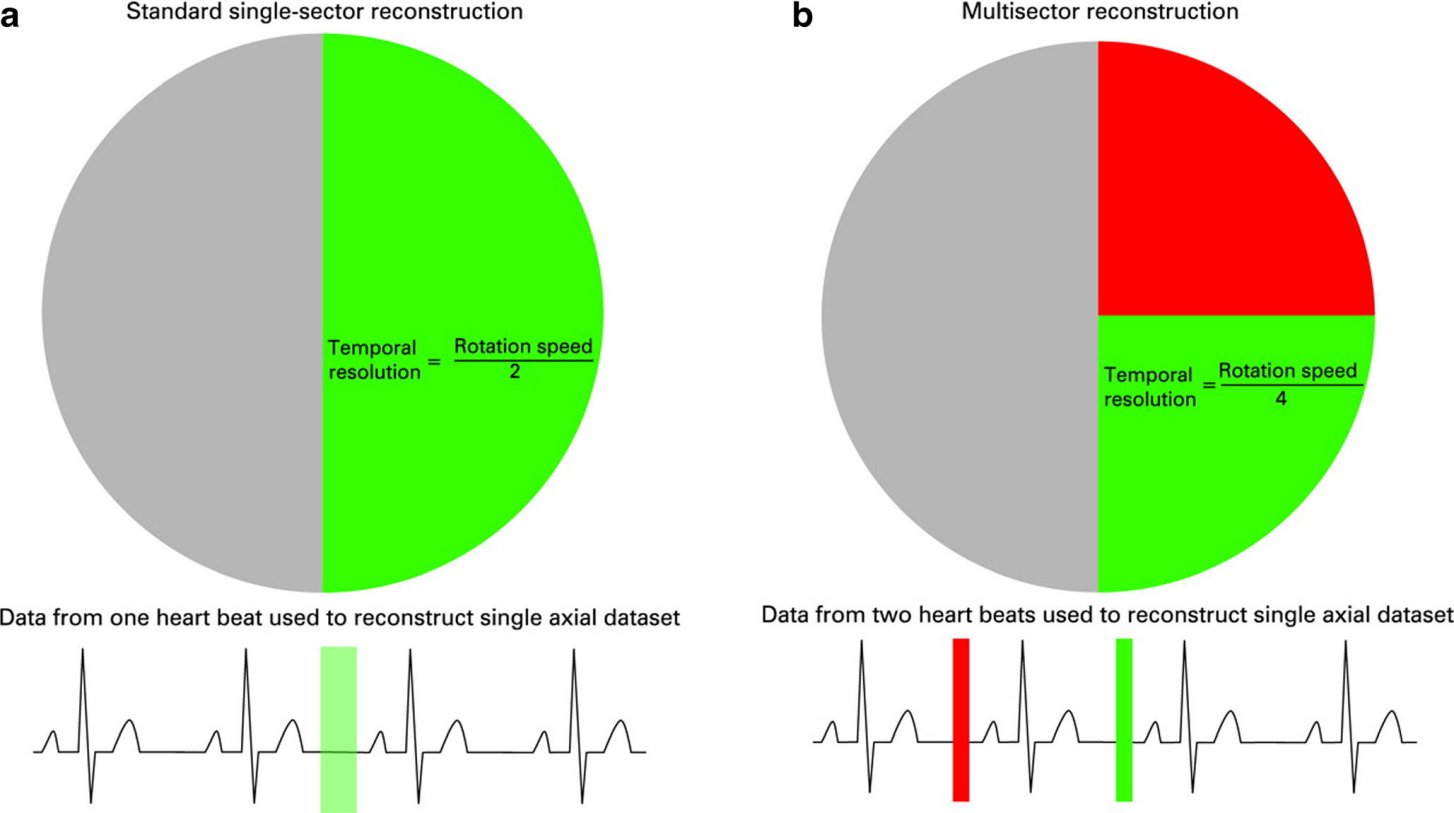
Schematic diagram of single versus multisegment reconstruction showing image acquisition (colored boxes) in mid-late diastole during one vs. two successive cardiac cycles. With multisegment reconstruction (right), image data from multiple cycles are combined to produce the image, thus improving the effective temporal resolution. The circle represents the angular range. Used with permission from Roberts et al. Heart. 2008 Jun;94 [6]:781–792

As shown in Fig. 5, MSR reduces the rotational arc of the gantry required by algorithmically combining data from 2 to 5 cardiac cycles into a single image. This approach, of use in both high and variable heart rates, can improve the temporal resolution of multidetector CT (MDCT) coronary angiography to 53 ms [20] but also introduces a new source of reduced image quality (e.g., blurring from imperfect alignment of adjacent data segments). In addition, MSR requires retrospective ECG-triggered acquisition and may require lower pitch, both of which may result in increased radiation dose to patients. When compared to ICA for the detection of significant (> 50%) stenosis in heart transplant recipients with elevated heart rates, MSR has a sensitivity, specificity, positive predictive value, and negative predictive value of 86%, 99%, 81%, and 99%, respectively [21••].

#### Intracycle Motion Correction Algorithms (MCAs)

MCAs determine coronary vessel position within the target phase of the cardiac cycle by utilizing both path and velocity information from adjacent phases within the same cardiac cycle [22]. Dependent on a single cardiac cycle, MCAs are considered less vulnerable to heart rate variability than MSR and may be associated with lower radiation doses as they can be applied to prospective ECG-triggered acquisitions [23]. A recent prospective, multicenter clinical trial showed that the MCA studied improved image quality and improved diagnostic performance for obstructive CAD on a per-vessel basis and on a per-subject basis in patients with a heart rate > 70 beats/min [24••].

#### Recent Clinical Data

A recent meta-analysis including data from 13 studies and 615 cardiac transplant recipients showed that currently available coronary CTA technology offers a reliable non-invasive alternative to ICA. In this study, patient-based analyses comparing coronary CTA versus ICA for the detection of significant CAV (> 50%) showed a mean weighted sensitivity of 94%, specificity of 92%, negative predictive value (NPV) of 99%, positive predictive value of 67%, and diagnostic accuracy of 94%, with a strong trend toward improved sensitivity and negative predictive value with 64-slice compared with 16-slice coronary CTA [25••].

Among studies included in this meta-analysis, Mittal et al. provided the largest prospective head-to-head comparison of coronary CTA and ICA for CAV screening [26]. They included 138 cardiac transplant patients undergoing routine ICA and concurrent coronary CTA for comparison at a mean of 12 years after transplant. With a single-source 64-slice scanner (~ 175 ms temporal resolution), at an average heart rate of 83 ± 4 bpm during image acquisition (no beta-blocker use), CTA image quality was diagnostic in 96% of patients and 98% of all coronary segments. By comparison to ICA for the diagnosis of CAV, CTA achieved a per-patient AUC = 0.88 (95% CI 0.82–0.94) for CAV with any stenosis, and an excellent AUC = 0.94 (95% CI 0.89–1.0) for CAV with ≥ 50% stenosis. None of the 61 patients with a normal coronary CTA had CAV on the basis of ICA, for an overall negative predictive value of 98–99%. Notably, 41% of included patients (*n* = 56) had an estimated GFR = 30–59 mL/min/1.73 m^2^, and no patients developed contrast induced nephropathy after CTA and/or ICA procedures.

### CT-Derived Fractional Flow Reserve May Better Identify Diffuse CAV

**Fig. 6:**
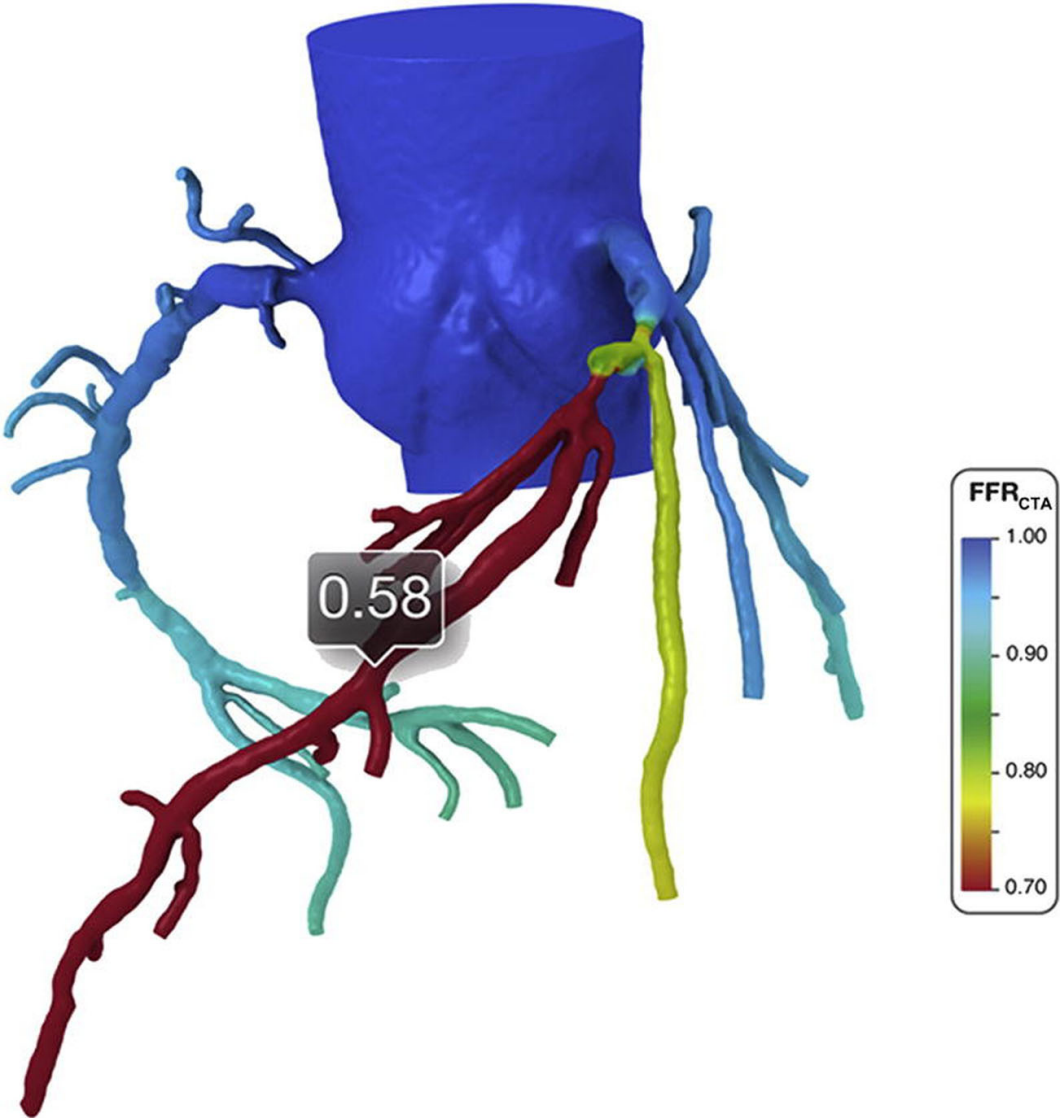
Coronary CTA-derived fractional flow reserve (CT-FFR) in a patient with serial lesions in the left anterior descending coronary artery. Color contours provide data on the distribution of CT-FFR throughout the coronary tree with numerical values obtainable at any location. Used with permission from Taylor et al. J Am Coll Cardiol. 2013 Jun 4;61 [22]:2233–41

Another recent technological advance associated with coronary CTA is CT-derived fractional flow reserve (CT-FFR). Analogous to FFR obtained from pressure wire pullback during diagnostic ICA, CT-FFR is designed to measure the hemodynamic effects of epicardial coronary artery stenosis. CT-FFR is computed using anatomic information and computational fluid dynamic modeling, and therefore requires source images of good quality [27]. As shown in Fig. 6, this application allows for quantification of CT-FFR values anywhere within the coronary tree. As such, CT-FFR offers the potential for hemodynamic assessment of diffuse concentric CAV in all three coronary territories without the inherent risk associated with multiple invasive pressure wire interrogations during ICA. While the clinical utility and cost-effectiveness of CT-FFR in heart transplant recipients has never been reported, clinical trials in non-heart transplant recipients have been encouraging. The NXT trial showed that CT-FFR provides high diagnostic accuracy and discrimination for the diagnosis of hemodynamically significant CAD and led to a marked increase in specificity when compared to standard coronary CTA [28]. In addition, the PLATFORM trial showed that utilization of CT-FFR was associated with a significantly lower rate of non-obstructive CAD at invasive angiography [29••]. In other words, coronary CTA with CT-FFR effectively triaged patients to minimize unnecessary ICA. Whether or not these clinical trial findings can be replicated specifically in heart transplant recipients remains unknown but worthy of prospective evaluation.

### Transitioning to Coronary CTA for CAV Surveillance: Potential Barriers

Despite the technological advances described above, potential “real-world” barriers may limit the viability of coronary CTA as a preferred modality for CAV surveillance in heart transplant recipients. First, health care facilities with the necessary hardware, software, and, most importantly, the cardiovascular imaging experts capable of correctly employing these technologies to perform high-quality coronary CTA in transplant patients remain scarce. Second, coronary CTA is more likely than ICA to identify cardiac and non-cardiac incidental findings that necessitate follow-up that may not ultimately improve patient care. The marginal cost and psychological burden that this imposes on patients and health care systems must be included in any determination of the cost-effectiveness of any coronary CTA-first surveillance strategy. Finally, other non-invasive diagnostic modalities are emerging as potential alternatives to ICA for CAV surveillance. In particular, myocardial flow reserve derived from myocardial perfusion positron emission (PET) has recently been shown to have both diagnostic and prognostic value for CAV evaluation in heart transplant recipients [30, 31].

### Conclusion

In summary, recent technological advances in coronary CTA allow for significantly improved image quality and diagnostic accuracy in patients with elevated heart rates such as heart transplant recipients. CT-FFR may add further value in this clinical scenario. Overall, coronary CTA now has equivalent diagnostic accuracy, offers more nuanced anatomic information, is inherently safer, and could be less costly than invasive coronary angiography. For these reasons, coronary CTA may now be a viable alternative to ICA for CAV surveillance in heart transplant recipients.
