# Correction to: Coronary CTA for Surveillance of Cardiac Allograft Vasculopathy

**DOI:** 10.1007/s12410-018-9476-y

**Published:** 2018-11-08

**Authors:** Nishant R. Shah, Ron Blankstein, Todd Villines, Hafiz Imran, Alan R. Morrison, Michael K. Cheezum

**Affiliations:** 1 Lifespan Cardiovascular Institute, Division of Cardiovascular Medicine, Dept. of Medicine, Brown University Alpert Medical School, Providence, RI USA; 2 Dept. of Medicine (Cardiovascular Division) and Radiology, Brigham and Women’s Hospital, Harvard Medical School, Boston, MA USA; 3 Dept. of Medicine, Cardiology Service, Walter Reed National Military Medical Center, Bethesda, MD USA; 4 Dept. of Medicine, Cardiology Service, Fort Belvoir Community Hospital, Ft. Belvoir, Fairfax County, VA USA

**Correction to: Current Cardiovascular Imaging Reports (2018) 11: 26**

10.1007/s12410-018-9467-z

The article **Coronary CTA for Surveillance of Cardiac Allograft Vasculopathy**, written by Nishant R. Shah, Ron Blankstein, Todd Villines, Hafiz Imran, Alan R. Morrison and Michael K. Cheezum, was originally published electronically on the publisher’s internet portal (currently SpringerLink) on 24 September 2018 without open access.

With the author(s)’ decision to opt for Open Choice the copyright of the article changed on November 2018 © The Author(s) 2018 and the article is forthwith distributed under the terms of the Creative Commons Attribution 4.0 International License (http://creativecommons.org/licenses/by/4.0/), which permits use, duplication, adaptation, distribution and reproduction in any medium or format, as long as you give appropriate credit to the original author(s) and the source, provide a link to the Creative Commons license and indicate if changes were made.

The original article has been corrected.

